# Creating life and the media: translations and echoes

**DOI:** 10.1186/s40504-018-0087-9

**Published:** 2018-08-20

**Authors:** Manuel Porcar, Juli Peretó

**Affiliations:** 1 Institute for Integrative Systems Biology I2SysBio (UV-CSIC), Parc Científic de la Universitat de València, C. Catedràtic José Beltrán 2, 46980 Paterna, Spain; 2 Darwin Bioprospecting Excellence SL, Paterna, Spain; 3 Department of Biochemistry and Molecular Biology, Universitat de València, Burjassot, Spain

**Keywords:** Synthetic life, Artificial life, Social media, History of biology, Popular science, Minimal cell

## Abstract

Synthetic biology is the engineering view on biotechnology that ultimately aims at fulfilling the quest of building an artificial cell. From the very first attempts of synthesizing life, this subject has made an impact on the media through, very often, misleading headlines and news. We review here the historical journalistic approach on synthetic biology and related disciplines, from the early twentieth century to the lastest achievements on designing protocells or genome reduction. However, it would be very naive to consider the research community and the media to be unidirectionally linked, with the latter being mere displayers (and disrupters) of the research “reality”. On the contrary, the research community has also received a strong influence from the media, as evidenced by statements from researchers, common metaphors and, even, a trend to unconsciously develop shared techno-social paradigms. We conclude that, beyond overstatements from researchers and journalists’ misunderstandings, both communities provide strong feedback to each other and, together, contribute to define the dream that synthetic biologists are aiming for.

## Introduction

Synthetic biology is a term that, during the last 15 years, has shown an exponential occurrence in scientific literature, and has been a flagship for funding agencies for innovative research and for emergent technologies. The term actually refers to several approaches that aim at synthesizing new forms of life in the laboratory, including the search for minimal genomes (or top down strategy), the development of complex chemical mixtures performing life-like activities (or bottom up approach), the expanding of the genetic code and the amino acid repertoire beyond the natural one (or xenobiology) or the extension of classical metabolic engineering into new and unnatural processes (Acevedo-Rocha 2016). In most projects, the ultimate aim is the implementation of new devices with practical applications (Morange 2009; Friedman and Ellington 2015; Nielsen and Keasling 2016), although some scientists also anticipate that the making of life through synthetic biology will increase our understanding of it (Benner 2003; Szathmáry 2004; Keller 2009). An underlying assumption for most synthetic biologists is that engineering principles (i.e. standardization, decoupling, modularity, abstraction, orthogonality) can be extrapolated to biological objects (Endy 2005; Ausländer et al., 2017), although there is a long tradition of criticisms to this strongly mechanistic view of life (Nicholson 2014; Porcar and Peretó 2016).

A wide group of scientists and engineers recognize themselves as members of a synthetic biology community, and global collaborative networks, new scientific journals, recurring meetings and international contests (like the international Genetically Engineered Machine –iGEM– competition) demonstrate that the label “Synthetic Biology” has been successful, although some voices raise concerns about the pertinence of the term. In short, science and the market have enthusiastically adopted the term but, as recognized by Nerlich and McLeod (2016) “Synthetic Biology is still not a topic of public interest”. Nevertheless, the prospect of an artificial life created in a laboratory has attracted the media’s interest for over a century. The terrifying possibility of animating dead matter was supported by several research lines, like galvanism, and was a mesmerizing attractor for many writers and journalist’s attention, as well as serving as an inspirational source 200 years ago for Mary W. Shelley’s masterwork “Frankenstein or the modern Prometheus”. For most scientists, however, the prospect of making life in the laboratory was a very difficult, yet reachable in a remote future, possibility. As it has been analyzed elsewhere, there were several scientists, convinced materialists working in diverse cultural contexts, that pursued a sincere effort to cross the border between the inorganic and the living worlds (Peretó 2016). In the first part of this work we present some historical cases illustrating that the quest for a deeper understanding of the nature and origin of life has been seen, by the popular press but also by some scientists, as attempts to artificially create life in a test tube. We also analyze here the bidirectional influences between scientists and communicators that shape the ambitions of the synthetic biology community.

## Chronicle of a synthetic life foretold

The similarities among the motivations behind some of the research programs in biology a century ago and the extant ambitions in the synthetic biology community are surprising, as well as the parallelisms between the media responses evoked by scientists’ achievements. The news coverage of early synthetic biology experiments was so persuasive that the general discussion, even among scientists, was not whether the creation of life would be possible, but rather when it would occur (Turney 1998; Keller 2002).

Among the pioneers of an engineering approach to the synthesis of life we recognize Jacques Loeb (Pauly 1987). He discovered artificial parthenogenesis and developed a coherent ideology on understanding life through the synthesis and complete control of it. For Loeb, living organisms were –literally, not metaphorically– chemical machines that, one day, would be fully manufactured in a laboratory (Loeb 1904), and hence for him “experimental abiogenesis” (or the original evolution of life from inorganic or inanimate substances) was the “goal of biology” (Loeb 1906). We could consider Loeb as a forerunner of the harder version of current synthetic biology, in which the engineering approach to biology is based on a materialistic and extremely mechanistic view of life and on Loeb’s proposal of knowing life through synthesis, rather than analysis (Deichmann 2009; Pauly 1987; Keller 2009; Keller 2002). Also, following Keller’s (2002) distinction –inspired by (Gruenberg 1911b)– of two senses of the term “artificial life”, Loeb represented the efforts of building biological explanations through the “artificial synthesis of life” –like the artificial induction of development in unfertilized eggs. However, other scientists, marginalized by the official history of biology, used the “synthesis of artificial life” as a model to study of the origin and nature of life. In retrospect, the achievements of scientists like Alfonso L. Herrera with his “Plasmogeny” (Herrera 1942; Ledesma-Mateos and Barahona 2003; Cleaves et al. 2014), John Burke with his “radiobes” (Burke 1905a; Burke 1905b; Campos 2015) or Stéphane Leduc with his impressive osmotic growths (Leduc 1910; Leduc 1912; Keller 2002) could be seen as eccentric efforts to understand the origin and nature of life, although they should be better contextualized as relevant episodes in the history of biological explanations, *en route* to the full secularization of the studies of living objects (Keller 2002; Keller 2009; Peretó and Català 2007; Peretó 2016).

John B. Burke investigated the effect of radium salts on standard microbiology culture media and described, at the beginning of twentieth century, the generation of what he named “radiobes”, which he thought were primitive biological forms (Burke 1905a; Burke 1905b; Peretó 2016). In a comprehensive study of Burke’s work, historian Luis Campos has collected and analysed the extensive coverage of radiobes in newspapers from both sides of the Atlantic (Campos 2015), showing that Burke’s and Loeb’s findings were commonly seen by the popular press as related works associated with the synthesis of artificial life. Although, for some journalists, Burke’s experiments implied that the creation of life was just a reasonable possibility for the near future, for others radiobes were an example of fully accomplished artificial life. Some headlines were as explicit as “Generation by radium: Cambridge professor reported to have produced artificial life” (*The New York Times*, July 16, 1905), although Burke never claimed that he had actually synthesized life (Campos 2015).

The antivitalistic position was a common feature of the early attempts to synthesize life, and this aspect was perfectly understood by strongly ideologized scientists, particularly, Catholic priests involved in scientific research and teaching (Peretó and Català 2012; Peretó and Català 2017). Nevertheless, for scientists involved in the pioneering work of disentangling the chemical complexity of cells, including Loeb himself (Loeb 1920; Pauly 1987; Deichmann 2012), the used feedstocks (typically mineral and inorganic components) and the results obtained (“colloidal precipitates”) were poor imitations of living phenomena, mainly because they lacked the “synthetic power of transforming small ‘building stones’ into the complicated compounds specific for each organism” which, according to Loeb, “is the ‘secret of life’ or rather one of the secrets of life” (Loeb 1916). This *metabolic failure* of Herrera’s, Burke’s, Leduc’s and some others’ chemical constructs was thus its Achilles’ heel to the eyes of the earliest biochemists –see, for instance, the criticisms to Leduc’s constructs by (Oparin 1938) and to Herrera’s experiments by (Oparin 1957). Nevertheless, these biochemists recognized that having a deep knowledge of the chemical complexity of cells was the only way to start the path of the synthetic approach, much like the intellectual and heuristic progression that occurred with synthetic organic chemistry, starting in the nineteenth century and pursuing, in a stepwise manner, the synthesis of increasingly complex molecules. Thus, for the incipient biochemists the early Synthetic Biology efforts were not completely meaningless but they were premature or merely naive (Loeb 1916; de Gregorio Rocasolano 1917; Rodríguez Carracido 1917). However, these criticisms contrasted with the popularity achieved by the artificial plants made *à la* Leduc, even decades after his publications, as reflected for instance in Thomas Mann’s passage in his novel “Doctor Faustus”, which recreates the atmosphere of surprise and contradictory feelings before the osmotic growths prepared by the father of the composer Adrian Lewerkühn.

Loeb was particularly well positioned to criticize those untimely attempts to synthesize life, given his rigorous physicochemical approach to living phenomena. With his discovery of artificial parthenogenesis in 1899, he was convinced that fertilization and development were no longer an issue of morphology but of physics and chemistry. He never regarded or presented the induction of egg development by changing the chemical nature of the solution as an artificial synthesis of life. That was the task for journalists with their sensationalist headlines: “Science nears the secret of life. Professor Jacques Loeb develops young sea urchins by chemical treatments. Discovery that reproduction by this means is possible a long step towards realizing the dream of biologists, ‘to create life in a test tube’” (*The Chicago Tribune*, November 19, 1899). Thus, Loeb likely represents the first case of an experimental scientist publicly exposed by newspapers with the label of the “creation of life” ambition (Pauly 1987), albeit not directly related to the study of the historical origin of life or the empirical reproduction of spontaneous generation, as was the case of Herrera, Leduc or Burke, but engaged in unveiling the ‘secret of life’.

The runaway excitement in the newspapers forced him to declare in the journal *Science*: “In view of the fact that a number of daily papers have printed reports concerning alleged or real experiments of mine I wish to state: 1. That none of the statements printed in the newspapers have been authorized by me. 2. That whatever I may have to say about my work will be published in scientific journals.” (Loeb 1901). But this announcement did not prevent more grandiose slogans: “Chemical creation of life” (*The New York Times*, March 1,1905), “Creates life by chemistry” (*The Chicago Tribune*, March 1, 1905), “Dr. Jacques Loeb […] has succeeded in demonstrating how life may be produced by artificial means” (*The New York Times*, December 3, 1913) (quoted by Turney 1998). The atmosphere was also favorable to fake news: “The Mexican consul in Trieste reports that Prof. Herrera, a Mexican scientist, has succeeded in forming a human embryo by chemical combination” (*The New York Times*, October 4, 1910). At any rate, Loeb was clearly uncomfortable with the way that newspapers treated his discoveries but, at the same time, his firm mechanist ideology led him to legitimate, as stated before, the synthesis of life as the “goal of biology” (Loeb 1906). Turney (1998) pointed out Loeb’s ambivalent attitude since, while opposing wild journalistic exaggerations on his research, he strongly encouraged young scientists with his mechanist and synthetic research program for biology. A possible explanation is that, in spite of proposing experimental abiogenesis as the ultimate aim in biology, he certainly disliked the possibility of his work being associated with the objects obtained by people like Leduc or Burke, presented by the popular press –and represented in popular culture– as truly instances of artificial living forms, but that found in Loeb one of their firmest detractors (Loeb 1916).

Writing in *Scientific American*, Benjamin C. Gruenberg established a clear distinction between the approaches of Leduc, Burke and others to artificial life, versus the experiments of Loeb and others on artificial parthenogenesis (Gruenberg 1911b; Gruenberg 1911a). “From time to time we are informed that the Riddle of the Universe has been solved by the artificial production of ‘life’ from non-living materials” affirms Gruenberg, “but each time we wondered how it was done, for a few days, and then find out that it wasn’t done at all” (Gruenberg 1911b). Echoing the biochemists’ criticism based on life chemical complexity (still largely unknown at that time) to the “creation of artificial life”, Gruenberg insisted: “The failure of scientists so far to produce ‘artificial life’ is not to be charged against the science of biology. Very few of the attempts to produce ‘artificial life’ have been made by biologists, who realize too well the complexity of the problems involved. The biologists will be satisfied for a number of years to come if they succeed merely in analyzing what goes on in a living cell, in terms of physical and chemical processes. From time to time they will attempt to imitate the structure or a process by means of a working model; but they will not speak of artificial life until they are quite sure of all the conditions that play a part in this most intricate of phenomena” (Gruenberg 1911b). As the following examples show, although Gruenberg did not mention how journalists would contribute to the construction of the popular vision of making life in the laboratory, his prediction about the scientists’ cautious approach to artificial life was, to say the least, naive.

Whilst the earliest synthetic biologists’ yearnings were eclipsed by the molecularization of biology, some scientists explored the border between inanimate and animated matter in the emergent discipline of virology. The crystallization of the mosaic tobacco virus (TMV) by Wendell M. Stanley (Stanley 1935) had an enthusiastic reception by both scientific circles and popular media. At that time, the dominant view was that viruses and genes were made out of protein. Retrospectively, and thanks to the accurate work of several historians of science, we are able to recognize the diversity of methodological issues associated to Stanley’s observations, the conceptual mistakes and intellectual biases in the interpretation of his experiments, and the role played by himself and his institution (the Rockefeller Institute at Princeton) in the construction of a “revolutionary discovery” (Kay 1986; Helvoort 1991; Creager 2002). At any rate, Stanley seemed comfortable with the sensationalism elicited by his work and, through interviews and newspaper headlines, he promoted his ambitions as a researcher and the image of a scientist working in an almost philosophical field, namely, the nature of viruses as chemicals inhabiting the “twilight zone of life” (Kay 1986). It is of note that, at that time, these infective agents were generally seen as intermediate stages between the simplest living organisms and inanimate matter (Kay 1986; Helvoort 1991; Summers 2014). Therefore, the crystallization of TMV was presented, not surprisingly, as a “life in the making” effort and that “in the light of Doctor Stanley’s discovery, the old distinction between life and death loses something of its validity” (*The New York Times*, 29 June, 1935, cited by (Kay 1986).

How were Stanley’s achievements perceived by more specialized observers? Barclay Moon Newman, columnist of the popular science journal *Scientific American*, also labeled the purported TMV crystals as discoveries in the “twilight zone between life and non-life” (Newman 1937). His commentary was on the significance of bacteriophages, independently discovered by Frederick W. Twort and Félix d’Herelle in 1915 and 1917, respectively (Podolsky 1996; Summers 1999), and Stanley’s observations on TMV (Stanley 1935) in the context of a general discussion on the nature of genes and enzymes. Newman asserts that “it has astonished the scientific world that a single molecule can be the causative organism of a disease. How can a crystal be made up of living molecules?”, since the ability to crystalize was generally accepted as a property that was exclusive of inanimate matter, crystals of the causative agent of a disease with the ability to reproduce when in contact with the right host, would represent “an organization at the threshold of life”, and, thus, “in it we discover how the stages of increasing complexity of atomic combination have at last scaled up to the realm of life”. In a grandiose context of cosmic evolution, well inserted in the tradition of a progressivist thinking (Podolsky 1996), Newman considers the recent discoveries on the “virus molecule” (i.e. TMV) and phages as indicative of intermediate steps of an ascending scale of complexity from “unorganized matter” to “the order of life”.

The interest of John B. S. Haldane in many different aspects of life, including discussions on its nature, origin and eventual synthesis (Dronamraju 2017), is well known. In a series of popular science articles in the *Sunday Chronicle*, Haldane considered some “Unsolved problems of science”, and one of the chosen topics was “Can we make life?” (Haldane 1940). When discussing the frontier between the living and non-living, Haldane examined Stanley’s crystallization of TMV and concluded that “here, then, is a chemical substance which may be kept in a bottle and shows no sign of life; but given the right food it can reproduce itself”. For him, “the gap between chemistry and life has been very much narrowed”, reflecting the enormous impact of Stanley’s experiments on influential scientists like Haldane, while critically opposing to the popular press sensationalism. Thus, accepting the protein nature of the virus, for Haldane it was only a matter of further technical development to achieve the artificial building up of a complete virus “within the next thirty years”. Incidentally, the total chemical synthesis of the polio virus genome was described by Cello et al. (2002). In brief, despite Stanley’s outstanding claims, Haldane still considered that life hadn’t been synthesized yet, and, going further in his discussion, he affirmed that “it may be that artificial life of a simple character will be made in the laboratory long before we understand the process going on inside the cells of more complicated animals and plants”. Interestingly, his vision clearly contrasts with the engineering ideal represented by the now very famous posthumous sentence by physicist Richard Feynman “what I cannot create I do not understand”, widely quoted by contemporary synthetic biologists. Could we build a cell without knowing every detail of its intimate functioning? This seems the case at least in the design of a bacterium with a minimal genome in which almost one third of the required genes for life are of unknown function (Hutchison 3rd et al., 2016).

It is worth noting that the opinions expressed by Newman (1937) and Haldane (1940) eloquently exemplify the enthusiastic reception of Stanley’s experiments by a part of the scientific community, but with a different emphasis about their deeper scientific implications. The discovery of bacteriophages was an illuminating observation for Haldane in the context of his pioneering paper about the origin of life, in which he refers to the work of Twort and d’Herelle (Haldane 1929). He accepted the bacteriophage as a possible “missing link” in the evolution from inert to living matter in the primitive Earth, but, as pointed out by Podolsky (1996), his proposal was closer to considering the bacteriophage as an heuristic model rather than a real evolutionary stage, since for Haldane life was too dynamic and complex to be reduced to a simple molecule. Thus, Haldane’s model evolved in parallel to scientific knowledge and, 25 years after his first proposal, he invoked a more elaborated scheme with “the enclosure of several different self-reproducing polymers within a semipermeable membrane” as “the critical event which may have best be called the origin of life” (Haldane 1954), definitively considering bacteriophages as an operational model for the earlier stages of life (Podolsky 1996). During the Wakulla Springs International Conference on the Origin of Life in 1963, Haldane revised his ideas on the natural emergence and artificial synthesis of life, suggesting that “the initial organism may have consisted of one so-called ‘gene’ of RNA specifying just one enzyme” catalyzing the required reactions for RNA replication and protein synthesis (Haldane 1965). In this way, Haldane contributed also to a scientific debate in the 1960s on the origin of the genetic material and its associated minimal functions, with proposals from several scientists, including Alexander Rich, Carl Woese, Leslie Orgel and Francis Crick (Lazcano 2012). Haldane was still convinced of the importance of the synthetic approach to life and proposed that “if we have not committed planetary suicide, some of us, or of the next generation, will try to make a living organism”. As he emphasized during the following discussion (transcribed in the proceedings of the meeting), he was more interested in determining the “specifications for a synthetic organism” than in a “deduction as to what the first organism was”. For him, in the mid-1960s, artificial life was still a legitimate, attainable, not yet realized scientific goal. Following a genocentric view of life, he suggested that “the first synthetic organisms may have been something like a tobacco mosaic virus, but including the enzyme or enzymes needed for its replication”, implying that the presence of any sort of “semipermeable membrane” in our test tube assays for synthetic organisms was, therefore, unnecessary and could differ from the primitive living stages.

As it is easy to understand, and Haldane’s charismatic contributions constitute a wonderful example, the discussions and the experimental approaches to the origin of life have always been a fertile field for artificial life imagination. The founding fathers of the discipline, Oparin and Haldane, were independently proposing the experimental approach to artificial living constructs as a way to evade mere speculations on this topic (Haldane 1929; Oparin 1938). But, surprisingly, even the simplest simulations, as the first experimental set to validate one of Oparin’s postulates –namely, the abiotic synthesis of life building blocks under primitive conditions– published by Stanley L. Miller (Miller 1953), elicited media enthusiasm but also raised concerns about the possibility to synthesize life in a chemical laboratory. Even Miller himself was astonished by the wide media coverage of his paper (Miller 1974). A Gallup poll performed after Miller’s experiment announcement delivered a majoritarian negative (78%) versus a slight affirmative support (9%) and 13% of “don’t know” to the question “would it be possible to create life in a test tube?”. So the skeptical headlines stated that “Many doubt science may create life” (*Los Angeles Times*, June 8, 1953).

A remarkable example of a scientific discovery mistaken for the synthesis of life is Arthur Kornberg and coworkers description of the synthesis of a phage DNA genome in a test tube by a purified bacterial enzyme with polymerase activity (Goulian et al., 1967). The synthetic DNA showed the same structure, sequence and infectivity as the natural viral genome. As Kornberg states in his autobiography (Kornberg 1991), he took personal care that the news office of his institution, Stanford University, would describe the work with accuracy but cautioning journalists during the press conference that the experiment had nothing to do with “synthesizing life in a test tube”. That same day, during a statement at the Smithsonian Institution, President Lyndon B. Johnson referred to the work and said “some geniuses at Stanford University have created life in the test tube!”. The next day, a frustrated Kornberg read all the newspaper stories about his work beginning with the presidential hyperbole (Kornberg 1991).

Biochemist John H. Northrop, who together with James B. Sumner established the protein nature of enzymes –both biochemists shared the Nobel Prize in Chemistry 1946 with Stanley–, was a former collaborator of Loeb and, as late as 1961, considered that artificial parthenogenesis experiments “are still, I believe, the nearest approach to the creation of life” (Northrop 1961). As a strict materialist-experimentalist in the best Loebian tradition, he emphasized that the “failure to create life in the laboratory has given rise to a number of unnecessary assumptions” including the existence of “some intangible force”. As an active investigator in bacteriophage research in the 1930s, Northrop concluded that, from a biochemical and metabolic point of view, viruses cannot be considered alive; otherwise, many virology experiments should be viewed as instances of creation of life (Northrop 1961). However, by advocating a minimalist notion of what life is –“a living system [is] one that can use energy to carry out the synthesis of more of itself”– he considered that the polymerase reaction described by Kornberg and coworkers “represents the simplest living system” (Northrop 1961). In that sense, Northrop’s position was closer to President Johnson’s than to Kornberg’s.

The seventies, eighties and nineties of the twentieth century were characterized by the explosion of molecular biology and biotechnology (Morange, 2003). In the last decade of the century, and with an unprecedently short delay, transgenic plants were created, commercialized and spread worldwide to become a very significant part of all the crops grown today (for a complete report, see ISAAA 2016). The development of biotechnology and related disciplines, namely metabolic engineering, during the last years of the past century, yielded impressive reports on synthetic biology *avant la lettre*; the best example being the so-called Golden Rice, a genetically engineered rice with a complete metabolic pathway leading to the synthesis of high levels of provitamin A. The Golden Rice was specifically made to prevent the severe symptoms of Vitamin A deficiency, and it was first published in *Science* in 2000 (Ye et al. 2000). It may somehow be considered as a shift of paradigm from biotechnology-issued GM plants towards synthetic-biology-issued ones, not because of the strict engineering principles being used, but because of the degree of sophistication of the artificially modified metabolic pathway (Porcar and Peretó 2012).

## Synthetic biology today: The return of the biomachinery

It has to be stressed, though, that contemporary, sensu *strictu* synthetic biology basically relates to research in biological engineering performed during the twenty-first century, and that this young –two decades-old– research field is characterized by a renaissance of the biomachine assumption together with an explosion of associated buzzwords (Peretó and Catalá 2007; de Lorenzo and Danchin 2008; Porcar and Peretó 2016), and by an active search and development of parallelisms between synthetic biology and industrial and electric engineering. This has created some conflicts among synthetic biology practitioners, since biologists and engineers tend to have contrasting views on the nature of life, its complexity and its amenability to rational design (Delgado and Porcar 2013). In general, the engineering view has prevailed and, as a consequence, both the scientific literature and the press releases on synthetic biology tend to show a mechanistic view on biotechnology.

One of the most famous quotations evidencing the purely engineering conception of the living matter is Drew Endy’s statement on emergent properties, a key feature of life: “Engineers hate complexity. I hate emergent properties. I like simplicity. I don’t want the plane I take tomorrow to have some emergent property while it’s flying” (Edge 2008). The message here is clear: machines are more trustworthy than organisms, and thus the latter have to be “machinised” to become reliable. The same author, in another interview a few years later, went further in the description of rational design of synthetic organisms by proposing what he considers a realistic –yet futuristic– way to build a biological laptop: “fill up the can with sawdust, add some programmed wood fungus (…) I come back a week later, and I shake out all the extra loose sawdust and spent materials, and out comes my new laptop” (Ananthaswamy 2014). Finally, in their provocative book “Regenesis: How Synthetic Biology will reinvent nature and ourselves”, Church and Regis (2012) describe a rather similar example: a plant so deeply engineered to develop into… a house (Church and Regis 2012). Both claims share a strikingly blurred boundary between the metaphor and the prediction. The point here is whether those public declarations have to be taken literally. How could engineered plant or fungal tissues develop into electric conductors, transparent screens or windows, mobile components, etc.? What would the fungal computer and the plant-house look like? Like a real laptop and a ready-to-move-in house or like a rough abacus and a giant pumpkin, respectively?

In 2010, a viable bacterial cell with a transplanted, chemically synthesized chromosome was reported in an article in *Science* entitled “Creation of a bacterial cell controlled by a chemically synthesized genome” (Gibson et al., 2010). In the press conference that followed, the leader of the research group, Craig Venter stated: “this is the first self-replicating species we’ve had on the planet whose parent is a computer”. That statement, and the use of the word “creation” to describe an artificial version (almost completely a copy) of a natural genome, raised many criticisms (Table 1) including a well-balanced editorial in *The Guardian* (Anonymous 2010). A wide consensus against the hype of these declarations did not prevent, however, the discovery making the headlines worldwide, most of which echoed Venter’s pitch by highlighting that the first synthetic cell had indeed been created.

**Table 1:** Several examples of headlines generated by J. C. Venter Institute achievements (milestones after JCVI website: https://www.jcvi.org/publications)

Milestone	Headline	Reference
Bacterial genome transplantation (Lartigue et al. 2007)	“Playing God. How scientists are creating life forms or biodevices that could change the world”	*Newsweek* June 3, 2007
First synthetic bacterial genome (Gibson et al. 2008)	“Playing God: the man who would create artificial life”	*Independent* January 25, 2008
First self-replicating synthetic cell (Gibson et al. 2010)	“And man made life. The first artificial organism and its consequences”	*The Economist* May 20, 2010
	“Scientist Craig Venter creates life for first time in laboratory sparking debate about ‘playing god’. Artificial life has been created in a laboratory for the first time by a maverick scientist.”	*The Telegraph* May 20, 2010
	“American scientist who created artificial life denies ‘playing God’. Craig Venter, the American biologist who has created artificial life in a laboratory for the first time, has defended himself against accusations he was ‘playing God’.”	*The Telegraph* May 21, 2010
First minimal cell (Hutchison 3rd et al., 2016)	“Breakthrough in synthetic biology is far from ‘playing God’. The creation of a cell with the minimal number of genes necessary for life is to be applauded - mostly for what it tells us about our ignorance.”	*New Scientist* March 30, 2016

Interestingly, this and other subsequent reports on artificially built genomes or chromosomes (Baker 2011; Richardson et al. 2017; Mercy et al. 2017) coincided in time with a peak of not only written news, but also images and cartoons dealing with synthetic biology. Domínguez et al. (2014) studied a set of cartoons on synthetic biology published in Europe in three languages and grouped them in five main blocks: mystic/religious (playing God); monstrous (Frankenstein-like); engineering (biomachine metaphors); descriptive (no clear positioning); and comical. Although most of the cartoons were classified as not inherently negative, the high rate of sensationalist ones –particularly those of the monstrous group– supported the authors’ advice that synthetic biologists should choose their metaphors more carefully (Domínguez et al. 2014). Interestingly, one of the co-founders of the discipline, Drew Endy, coauthored with Isadora Deese a cartoon, drawn by Chuck Wadey and published in *Nature* five years before Venter’s high-profile breakthrough. The cartoon, entitled “Adventures in Synthetic Biology” describes how a kid discovers the tools to engineer organisms. This is an uncommon and commendable dissemination effort in a field in which a flow of information with the public is much needed, although its publication in a scientific journal certainly restrained the target audience of the message. Interestingly, the ETC group, which describes synthetic biology as “extreme engineering”, prepared in 2009 a comic to denounce the risks of synthetic biology, but it also displayed many similarities with later publication in *Nature*, since it also included similar representations of engineered DNA being injected into cells, which could consequently mutate (http://www.etcgroup.org/sites/www.etcgroup.org/files/cartoons/etcventertoons_story_of_lg.jpg). For an uninformed reader, it is difficult to identify which of the cartoons described above has been made by pro- or anti-synthetic biology members. In fact, the comic in *Nature* shows far more spectacular –and frightening– unexpected effects of bioengineering than the anti-synthetic biology cartoon.

Images, in cartoons or elsewhere, can be powerful visual metaphors, and synthetic biology is the paradise of metaphors. Biomachines, chassis, devices, circuits, factories… the list is endless to the point that it is often difficult to identify whether a term is used either in a metaphoric or realistic sense (de Lorenzo 2011). The use and effects of metaphors has recently been reviewed by Boldt (2016; and this thematic issue) and by McLeod and Nerlich (2017), the latter arguing (italics in the original text) that: “it is important to think about metaphors because they are not only used to *explain the world*, but they also affect how we *think about the world*” and thus claim for a systematic study “of the normative implications, and associated moral and ethical assumptions”. This leads us to the complex issue of the communication, in the broadest sense, between scientists and society, and between society and scientists. The media are somehow intermediate actors, and their view on synthetic biology is, in general, positive, in contrast with the less consensus in the views among policy makers, as it has been reported in the case of Dutch leaders (Ancillotti et al. 2016).

As we have discussed in the present article, the history of synthetic biology is rooted in the quest of producing artificial life, a common *leitmotif* of many cultures at all times. And this subject has always been –and still is– of the highest interest for the public. In the last century and, in particular, in the last two decades, scientific discoveries dealing with the manipulation of the living have had a constant, although at least partially unconscious, crosstalk of information between practitioners and the public, which has mainly taken place in the form of headlines, metaphors, images or very simple “informational pills” (almost always either false or largely inexact such as “artificial life has been created”). In this crosstalk, the responsibility of scientists towards the public is obvious, but their sensitivity to the feedback they perceive from society cannot be ignored either, and some historical cases, as Loeb’s and Stanley’s relationship with social media, eloquently illustrate such feedback. The intrinsic socio-cultural differences between scientists/engineers and the rest of the society cannot be erased, but the extreme simplification merged with hype of some statements does not contribute towards a transparent communication channel. Instead, a co-creation scenario would be very desirable, in which decisions on at least the final goals as well as technical red lights of bioengineering were agreed by all relevant stakeholders. In other words, rather than a crosstalk based on press releases and headlines, co-development of goals and tools (including metaphors) should be at least considered.

The development of a co-creation scenario in synthetic biology is a particularly challenging issue. It has previously been reported how semantic issues affect public perception of new technologies, the reaction being very different toward terms such as “GMOs” versus “Genetic Engineering”, for example (Verseux et al. 2016). Likewise, synthetic biologists are not necessarily good at estimating the knowledge of non-biologists (Verseux et al. 2016). These two issues may obviously distort communication and, combined with the abuse of metaphors described above, may certainly difficult the development of a fruitful co-creation scenario. In fact, the broadly acknowledged demand of a responsible research and innovation RRI-based integration of a broad range of social actors, including synthetic biology practitioners and social science and humanities scholars, has been proposed to require to move out from the “comfort zone” of the actors involved and to be developed based on an empiric approach (Delgado and Åm 2018; Delgado and Åm, 2018).

As a conclusion, the authors cannot resist the temptation of contributing to the challenging debate on the communication in synthetic biology by proposing a metaphor. A communication event resembles a call, in the sense of a shout. The sound can be sent back by obstacles and be received modified or distorted. On many occasions, it can also be received with different intensities and distortions. The dialogue between practitioners of synthetic biology and the public is thus not a frank and direct translation, but much like one in which actors are distant, and reach each other with echoes, a distortion of the own and others’ past statements. Those resonances are not under control of the emitters any more but determine the current perception as well as the fate of synthetic biology.

## References

[CR1] Acevedo-Rocha CG, Hagen K, Engelhard M, Toepfer G (2016). The synthetic nature of biology. Ambivalences of creating life. Societal and philosophical dimensions of synthetic biology.

[CR2] Ananthaswamy A.. Rewiring nature with synthetic biology. Discover, October issue. 2014. http://discovermagazine.com/2014/oct/17-natures-technician. Accessed 25 Feb 2018.

[CR3] Ancillotti M, Rerimassie V, Seitz SB, Steurer W (2016). An update of public perceptions of synthetic biology: still undecided?. NanoEthics.

[CR4] Anonymous. Synthetic cells: it’s life, but not as we know it The Guardian 2010 22 May. https://www.theguardian.com/commentisfree/2010/may/22/craig-venter-synthetic-life-editorial. Accessed 25 Feb 2018.

[CR5] Ausländer S, Ausländer D, Fussenegger M (2017). Synthetic biology-the synthesis of biology. Angew Chem Int Ed.

[CR6] Baker M (2011). Synthetic genomes: the next step for the synthetic genome. Nature.

[CR7] Benner SA (2003). Synthetic biology: act natural. Nature.

[CR8] Boldt J (2016). Metaphors, worldviews, ethics, and law.

[CR9] Burke JB (1905). On the spontaneous action of radio-active bodies on gelatin media. Nature.

[CR10] Burke JB (1905). On the spontaneous action of radium on gelatin media. Nature.

[CR11] Campos LA (2015). Radium and the secret of life.

[CR12] Cello J, Paul AV, Wimmer E (2002). Chemical synthesis of poliovirus cDNA: generation of infectious virus in the absence of natural template. Science.

[CR13] Church G, Regis E (2012). Regenesis. How synthetic biology will reinvent nature and ourselves.

[CR14] Cleaves HJ, Lazcano A, Ledesma Mateos I, Negrón-Mendoza A, Peretó J, Silva E (2014). Herrera’s “Plasmogenia” and other collected works: early writings on the experimental study of the origin of life.

[CR15] Creager ANH (2002). The life of a virus. Tobacco mosaic virus as an Exprimental model, 1930–1965.

[CR16] de Gregorio Rocasolano A (1917). Estudios Químico Físicos Sobre La Materia Viva.

[CR17] de Lorenzo V (2011). Beware of metaphors: chasses and orthogonality in synthetic biology. Bioengineer Bugs.

[CR18] de Lorenzo V, Danchin A (2008). Synthetic biology: discovering new worlds and new words. EMBO Rep.

[CR19] Deichmann U (2009). Chemistry and the engineering of life around 1900: research and reflections by Jacques Loeb. Biol Theor.

[CR20] Deichmann U (2012). Crystals, colloids, or molecules? Early controversies about the origin of life and synthetic life. Persp Biol Med.

[CR21] Delgado A, Åm H (2018). Experiments in interdisciplinarity: responsible research and innovation and the public good. PLoS Biol.

[CR22] Delgado A, Porcar M (2013). Designing de novo: interdisciplinary debates in synthetic biology. Syst Synth Biol.

[CR23] Domínguez M, Mateu A, Torgersen H, Porcar M (2014). Cartoons on bacterial balloons: scientists’ opinion on the popularization of synthetic biology. Syst Synth Biol.

[CR24] Dronamraju K (2017). Popularizing science. The life and work of JBS Haldane.

[CR25] Edge (2008). A talk with Drew Endy.

[CR26] Endy D (2005). Foundations for engineering biology. Nature.

[CR27] Friedman DC, Ellington AD (2015). Industrialization of biology. ACS Synt Biol.

[CR28] Gibson DG, Benders GA, Andrews-Pfannkoch C, Denisova EA, Baden-Tillson H, Zaveri J (2008). Complete chemical synthesis, assembly, and cloning of a mycoplasma genitalium genome. Science.

[CR29] Gibson DG, Glass JI, Lartigue C, Noskov VN, Chuang RY, Algire MA (2010). Creation of a bacterial cell controlled by a chemically synthesized genome. Science.

[CR30] Goulian M, Kornberg A, Sinsheimer RL (1967). Enzymatic synthesis of DNA, XXIV. Synthesis of infectious phage phi-X174 DNA. Proc Natl Acad Sci U S A.

[CR31] Gruenberg BC (1911). Artificial life. II. Making the non-living do the work of the living. Sci Am.

[CR32] Gruenberg BC (1911). The creation of ‘artificial life’. The making of living matter from non-living. Sci Am.

[CR33] Haldane, JBS. 1929. The origin of life. Rationalist Annual, 3–10.

[CR34] Haldane JBS (1940). Unsolved problems of science: IV. Can we make life?. Keeping cool and other essays.

[CR35] Haldane JBS (1954). The origins of life. New Biol.

[CR36] Haldane JBS, Fox SW (1965). Data needed for a blueprint of the first organism. The origins of Prebiological systems and their molecular matrices.

[CR37] Herrera AL (1942). A new theory of the origin and nature of life. Science.

[CR38] Hutchison CA 3rd, Chuang RY, Noskov VN, Assad-Garcia N, Deerinck TJ, Ellisman MH, et al. Design and synthesis of a minimal bacterial genome. Science. 2016;351(6280) aad6253 10.1126/science.aad6253 27013737

[CR39] ISAAA (International Service for the Acquisition of Agri-Biotech Applications) 2016. http://www.isaaa.org/resources/publications/briefs/52/download/isaaa-brief-52-2016.pdf. Accessed 25 Feb 2018.

[CR40] Kay LE (1986). WM Stanley’s crystallization of the tobacco mosaic virus, 1930-1940. Isis.

[CR41] Keller EF (2002). Making sense of life. Explaining biological development with models, metaphors, and machines.

[CR42] Keller EF (2009). Knowing as making, making as knowing: the many lives of synthetic biology. Biol Theor.

[CR43] Kornberg A (1991). For the love of enzymes. The odyssey of a biochemist.

[CR44] Lartigue C, Glass JI, Alperovich N, Pieper R, Parmar PP, Hutchison CA (2007). Genome transplantation in bacteria: changing one species to another. Science.

[CR45] Lazcano A (2012). The biochemical roots of the rna world: from zymonucleic acid to ribozymes. Hist Phil Life Sci.

[CR46] Ledesma-Mateos I, Barahona A (2003). The institutionalization of biology in Mexico in the early 20th century. The conflict between Alfonso Luis Herrera (1868–1942) and Isaac Ochoterena (1885–1950). J Hist Biol.

[CR47] Leduc S (1910). Théorie Physico-Chimique de La Vie et Générations Spontanées.

[CR48] Leduc S (1912). La Biologie Synthétique.

[CR49] Loeb J (1901). Unauthorized newspaper reports. Science.

[CR50] Loeb J (1904). The recent development of biology. Science.

[CR51] Loeb J (1906). The dynamics of living matter.

[CR52] Loeb J (1916). The organism as a whole. From a physicochemical viewpoint.

[CR53] Loeb J (1920). The proteins and colloid chemistry. Science.

[CR54] McLeod C, Nerlich B (2017). Synthetic biology, metaphors and responsibility life Sci. Soc Policy.

[CR55] Mercy G, Mozziconacci J, Scolari VF, Yang K, Zhao G, Thierry A (2017). 3D organization of synthetic and scrambled chromosomes. Science 355: eaaf4597.

[CR56] Miller SL (1953). A production of amino acids under possible primitive earth conditions. Science.

[CR57] Miller SL, Neyman J (1974). The first laboratory synthesis of organic compounds under primitive conditions. The heritage of Copernicus: theories “pleasing to the mind”.

[CR58] Morange M (2003). Histoire de la biologie moléculaire.

[CR59] Morange M (2009). A critical perspective on synthetic biology 1. Introduction: the rise of synthetic biology. HYLE.

[CR60] Nerlich B, McLeod C (2016). The dilemma of raising awareness ‘responsibly.’. EMBO Rep.

[CR61] Newman BM (1937). The smooth slide up to life. Sci Am.

[CR62] Nicholson DJ (2014). The machine conception of the organism in development and evolution: a critical analysis. Stud Hist Phil Biol Biomed Sci.

[CR63] Nielsen J, Keasling JD (2016). Engineering cellular metabolism. Cell.

[CR64] Northrop JH (1961). Biochemists, biologists, and William of Occam. Annu Rev Biochem.

[CR65] Oparin AI (1938). The origin of life.

[CR66] Oparin AI (1957). The origin of life on the earth.

[CR67] Pauly PJ (1987). Controlling life: Jacques Loeb and the engineering ideal in biology.

[CR68] Peretó J (2016). Erasing borders: a brief chronicle of early synthetic biology. J Mol Evol.

[CR69] Peretó J, Català J (2007). The renaissance of synthetic biology. Biol Theor.

[CR70] Peretó J, Català J (2012). Darwinism and the origin of life. Evol Edu Outreach.

[CR71] Peretó J, Català J. A reconciliation with Darwin? Divergent views on evolutionism in Erich Wasmann and Jaime Pujiula. Biologists and Jesuits Mètode Sci Stud J. 2017;7 10.7203/metode.7.7996.

[CR72] Podolsky S (1996). The role of the virus in origin-of-life theorizing. J Hist Biol.

[CR73] Porcar M, Peretó J (2012). Are we doing synthetic biology?. Syst Synth Biol.

[CR74] Porcar M, Peretó J (2016). Nature versus design: synthetic biology or how to build a biological non-machine. Integr Biol.

[CR75] Richardson SM, Mitchell LA, Stracquadanio G, Yang K, Dymond JS, DiCarlo JE (2017). Design of a synthetic yeast genome. Science.

[CR76] Rodríguez Carracido J (1917). Los fundamentos de la bioquímica. Bol R Soc Esp Hist Nat.

[CR77] Stanley WM (1935). Isolation of a crystalline protein possessing the properties of tobacco mosaic virus. Science.

[CR78] Summers WC (1999). Félix d’Herelle and the origins of molecular biology.

[CR79] Summers WC (2014). Inventing viruses. Annu Rev Virol.

[CR80] Szathmáry E (2004). From biological analysis to synthetic biology. Curr Biol.

[CR81] Turney J (1998). Frankenstein’s footsteps. Science, genetics and popular culture.

[CR82] van Helvoort T (1991). What is a virus? The case of tobacco mosaic disease. Stud Hist Phil Sci.

[CR83] Verseux C, Acevedo-Rocha CG, Chizzolini F, Rothschild LJ (2016). Misconceptions of synthetic biology: lessons from an interdisciplinary summer school. NanoEthics.

[CR84] Ye X, Al-Babili S, Klöti A, Zhang J, Lucca P, Beyer P, Potrykus I (2000). Engineering the provitamin a (beta-carotene) biosynthetic pathway into (carotenoid-free) rice endosperm. Science.

